# Multimethod assessment of design, mechanical and metallurgical characteristics of ProTaper Ultimate, TruNatomy Glider, ProGlider, Hyflex EDM and WaveOne Gold Glider glide path instruments

**DOI:** 10.1007/s10266-025-01135-z

**Published:** 2025-06-17

**Authors:** Tuğba Türk, Cangül Keskin, Doğan Çetin, Özlem Sivas Yılmaz

**Affiliations:** 1 Department of Endodontics, Faculty of Dentistry, Ege University, İzmir, Türkiye; 2 Department of Endodontics, Ondokuz Mayıs University, Samsun, Türkiye; 3 Private Practice, İzmir, Türkiye; 4 Department of Endodontics, Bolu Abant İzzet Baysal University, Bolu, Türkiye

**Keywords:** Cyclic fatigue, Differential scanning calorimetry, Hyflex OGSF, Glide path, Microhardness, ProTaper Ultimate, Torsional resistance

## Abstract

Nickel–titanium (NiTi) glide path instruments have been developed to enhance performance, but their mechanical and metallurgical properties vary significantly among different systems. This study compared the design, mechanical, and metallurgical properties of six nickel–titanium (NiTi) glide path instruments using a multimethod approach. A total of 420 ProGlider (PG) (16/.02–.06), WaveOne Gold Glider (WOGG) (15/.02–.06) and glide path files from the ProTaper Ultimate (PTU Slider) (16/.02–.06), TruNatomy (TNG) (17/.02), and Hyflex EDM (HF EDM) 10.05 and 15.03 systems were analyzed. Design features were assessed using a dental operating microscope and scanning electron microscopy (SEM). Mechanical properties, including static and dynamic cyclic fatigue, torsional resistance, bending resistance, and buckling resistance, were tested following standardized protocols. Metallurgical properties were evaluated through differential scanning calorimetry (DSC), SEM, energy dispersive spectrometry (EDS), and Vickers microhardness testing. Statistical analyses were performed using the Shapiro–Wilk test to assess normality, followed by appropriate parametric or non-parametric tests, including ANOVA with post-hoc Tukey and Kruskal–Wallis *H* tests with Bonferroni correction (*α* = 0.05). Design analysis revealed no major defects or deformations. WOGG and PG exhibited the highest cyclic fatigue resistance, while HF EDM 15.03 had the greatest torsional resistance. PTU Slider demonstrated the highest bending and buckling resistance. Metallurgical analysis indicated differences in phase transformation behavior, with TN Glider showing a two-stage phase transformation. Significant differences were observed among the tested instruments in terms of design, mechanical properties, and metallurgical characteristics. The multimethod approach provided a comprehensive understanding of these instruments, highlighting their suitability for clinical use based on specific mechanical and metallurgical properties. This multimethod analysis highlights significant differences in cyclic fatigue, torsional resistance, and flexibility among glide path instruments. WOGG and PG may be preferred for curved canals due to their superior fatigue resistance, HF EDM 15.03 for calcified canals requiring higher torsional strength, and PTU Slider for cases needing increased resistance to bending and buckling.

## Introduction

A glide path is the initial, smooth, and reproducible pathway that allows larger instruments to follow the same trajectory from the orifice to the apical foramen [1]. Establishing a glide path reduces procedural errors, such as instrument fracture, canal transportation, and ledging, while also minimizing postoperative pain [2–5]. Glide path preparation is particularly important when initial canal negotiation is challenging, such as in narrow, curved, or partially calcified canals. In addition, glide path instruments play a critical role in cases involving moderate to severe canal curvature, highly sclerotic dentin, or previously untreated root canals, where careful negotiation is essential for safe and efficient shaping [4, 6, 7].

The glide path preparation consists of two sequential steps known as micro and macro glide path preparation [6]. The micro glide path, often necessary in curved and narrow canals, is typically created manually using pre-curved stainless-steel files. In contrast, a macro glide path can be prepared either manually or with engine-driven NiTi instruments [6–8]. Whether used in manual or engine-driven techniques, glide path instruments are the first to shape narrow canals and acute apical curvatures. As a result, they are subjected to a combination of torsional stress and cyclic fatigue, both of which can lead to instrument fracture if the files lack sufficient mechanical strength. To reduce stress accumulation during glide path preparation, earlier systems utilized multiple files for incremental enlargement. However, advancements in metallurgy and design have led to the development of single-file glide path systems with larger tapers and varied kinematics. Rotary glide path instruments allow efficient dentin removal and smooth canal negotiation but are more prone to torsional failure. In contrast, reciprocating glide path instruments reduce torsional stress accumulation, enhance cyclic fatigue resistance, and lower the risk of instrument fracture [6].

Advances in NiTi metallurgy, including control memory alloys and electrical discharge machining (EDM), have improved the cyclic fatigue resistance and torsional strength of Hyflex (HF) instruments [9, 10]. The classical Hyflex EDM system includes a glide path file with a #10.05 size, while the newly introduced opener, glider, shaper and finisher (OGSF) sequence features a glide path instrument with a #15.03 size [11]. The OGSF sequence keeps the variable cross sections along the instrument but updates the glide path file with greater size and reduced taper in an attempt to provide a shorter learning curve while shifting from coronal enlargement to full-length shaping instruments [11].

WaveOne Gold Glider (WOGG) was among the first reciprocating glide path instruments and is associated with reduced preparation time and greater cutting efficiency [12, 13]. It is manufactured using gold-wire heat treatment and has a variable taper between 2 and 6%, with a tip size of #15 and a parallelogram cross-sectional shape [14]. The TruNatomy system is specifically designed for minimal invasive procedures with a maximum flute diameter of 0.8 mm and features a glide path instrument TN Glider (TNG) with a #17 tip size and parallelogram cross section [15].

The ProTaper Ultimate (PTU) represents the latest advancement in the PT family. Since each file in a shaping system has a specific function, the metallurgy and dimension of each file in PTU files are uniquely designed to balance flexibility, cutting efficiency, and strength. This allows instruments with varied mechanical properties to strive to balance flexibility, cutting efficiency and strength [16]. The glide path file of PTU, the Slider, is made from M-wire and has a tip size of #16 and a variable taper of 2–6%, similar to the ProGlider (PG), but exhibits parallelogram cross-sectional shape.

Understanding the mechanical properties of glide path files is crucial for selecting instruments that can withstand torsional and cyclic stresses, thereby minimizing procedural errors [3, 6]. In addition, the quality of the glide path directly impacts the success of subsequent cleaning and shaping procedures. However, mechanical tests only reflect a single aspect of an instrument’s performance and may provide limited information to clinicians [17]. A multimethod assessment of NiTi instruments incorporates various techniques to evaluate their design, metallurgical properties and mechanical performance [17]. This comprehensive approach offers a more complete understanding of the instrument’s behavior under different conditions. To date, no study has evaluated the design, metallurgical characteristics, **or** mechanical properties of the novel glide path instruments of recent systems, such as TNG, HF OGSF**,** and PTU. Therefore, this study aimed to conduct a multimethod analysis of PTU Slider, PG, TN Glider, WOGG, HF EDM 10.05 and 15.03 instruments. The null hypothesis was there would be no significant differences in design, mechanical and metallurgical properties between the glide path instruments.

## Materials and methods

**Fig. 1:**
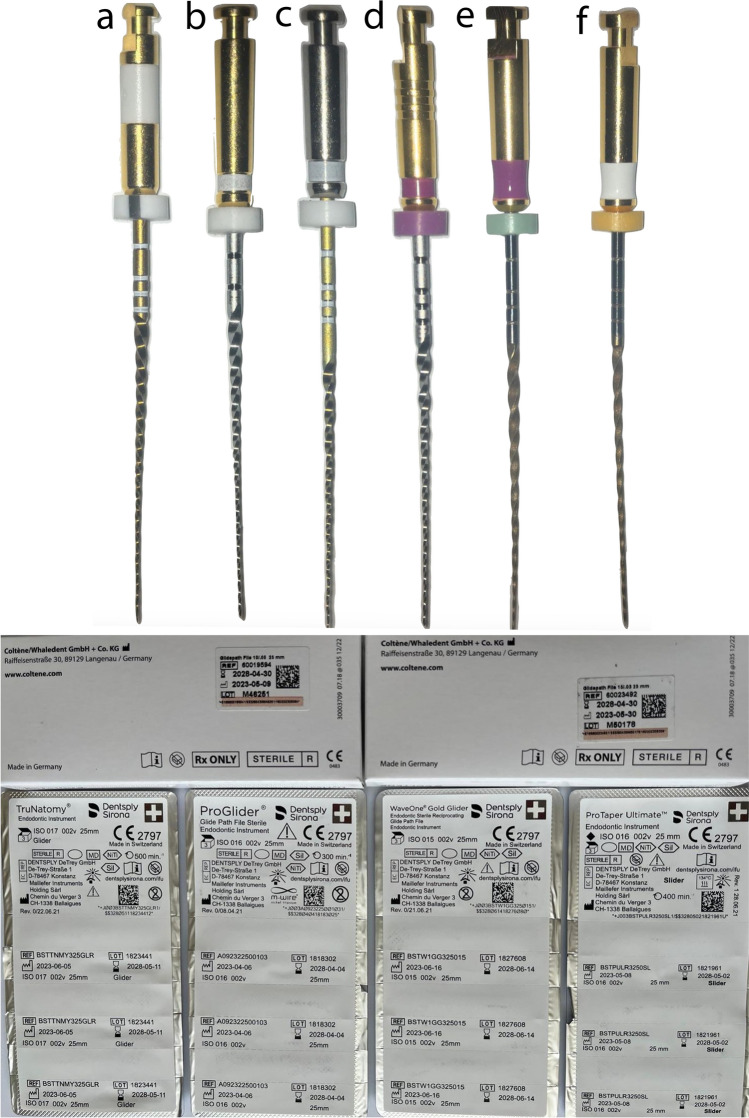
Macroscopic view of the tested instruments **a** WaveOne Gold Glider, **b** ProGlider, **c** TruNatomy Glider, **d** ProTaper Ultimate Slider, **e** Hyflex EDM 10.05, and **f** Hyflex EDM 15.03 with their packages showing LOT numbers and manufacturer information

A total of 420 new ProTaper Ultimate (PTU) Slider (Dentsply, Sirona, Ballaigues, Switzerland), ProGlider (PG) (Dentsply, Sirona), TruNatomy (TN) Glider (Dentsply, Sirona), WaveOne Gold Glider (WOGG) (Dentsply Sirona), and HyFlex EDM (HF EDM) 10.05 and 15.03 instruments (Coltene-Whaledent AG, Allstetten, Switzerland) (25 mm length) were collected (*n* = 70 per group) (Fig. 1).

### Design analysis

Four randomly selected instruments from different blisters of each system were and examined under a dental operation microscope with a magnification of 8.8× (Zumax OMS 3200, Zumax Medical Co., Ltd., China) to assess helical angles (calculated using an average of six most coronal measurements), the number of active blades, tip geometry and presence of major defects or deformation, such as distorted or twisted blades. The position of measuring lines was measured on 10 instruments using a digital caliper.

**Fig. 2:**
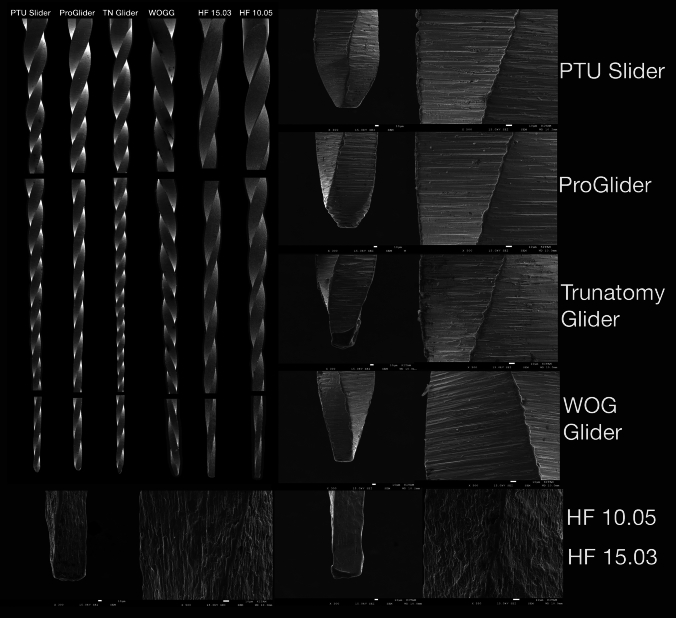
Scanning electron microscopic images of the tested glide path instruments showing the entire working part, blade and tip designs (× 16 and × 300 magnification) and surface finishing (× 500 magnification)

For surface characterization, scanning electron microscopy (JEOL JSM-7001F, Tokyo, Japan) was used to evaluate the spiral symmetry, tip and surface finishing, and cross-sectional shape at × 16, × 300 and × 500 magnifications (Fig. 2).

### Mechanical analysis

#### Cyclic fatigue resistance

For dynamic and static cyclic fatigue tests, the minimum specimen number was determined based on a priori power analysis using the effect size of a previous study (3.55), *F* test family (ANOVA; fixed effects, omnibus, one way) with an alpha-type error of 0.05 and power beta of 0.99 [18] in G*Power (G*Power for Mac v3.1; Heinrich-Heine-University, Düsseldorf, Germany). A total of 9 samples per group were determined as the required sample size to show significant differences. Twelve samples per group were collected after examining under DOM for any major defects (*n* = 12). No file was discarded.

Dynamic cyclic fatigue testing was carried out in a stainless-steel artificial canal with a curvature angle of 90° and curvature or radius of 2 mm. Intracanal temperature was simulated by covering the testing block with a heating wire (37 °C). The thermostat and thermocouples were used to measure intra-block temperature. The synthetic oil (WD-40; Milton Keynes, UK) was applied for lubrication of the artificial canal. All instruments were operated with a speed of 1-mm/sec and a total of 3 mm axial movement until fracture occurred. Axial displacement was controlled by a holder moving at a predetermined speed between two sensors, ensuring precise and consistent axial motion throughout the test.

Static cyclic fatigue testing was performed within a hot water bath at 37 °C. The same fatigue testing block having an angle of curvature of 90° and a radius of curvature of 2 mm was used as described in a previous study [19].

WOGG instruments were used with a VDW Silver endodontic motor (VDW; Munich, Germany) in “WaveOne ALL”, while PG instruments were operated with 300 revolutions per minute (rpm) and 5 Ncm torque. TN Glider instruments were operated at 500 rpm and 1.5 Ncm, the PTU Slider was operated at 400 rpm and 4 Ncm, and HF instruments were used at 300 rpm and 1.8 Ncm.

#### Torsional resistance test

For the torsional resistance test, sample size calculation indicated 12 samples were required as the minimum ideal size required to observe the same effect (1.62) [9]. The torsional strength of each instrument was determined as described in a previous study [9] by clamping at 3 mm from the instrument tip (*n* = 12). The instruments were then rotated in a clockwise direction at 2 rpm until separation, except the WOGG was rotated in **a** counter**-**clockwise direction. The ultimate torsional strength (Ncm) and the angle of rotation at failure (°) were monitored.

#### Bending tests

For the bending resistance test, an a priori sample size test indicated 3 samples per group as the minimum ideal size required to observe the same effect (1.96) [20]. The bending resistance was performed on 10 specimens per group using a universal testing machine (Instron Corp., Canton, MA, USA), with a 20 N load cell attached with a speed of 15 mm/min. The force at which the instrument underwent an elastic displacement of 45° was recorded.

#### Buckling tests

For buckling resistance, sample size analysis indicated that 2 samples per group were required as the minimum sample size (3.39). Ten specimens per group were used. The instrument shaft was mounted on the universal testing machine and the tip was compressed under a stainless-steel surface. The universal testing machine applied 20 N with a speed of 1 mm/s axillary until the elastic displacement of 1 mm, which the force was recorded.

### Metallurgical analysis

#### SEM analysis

**Fig. 3:**
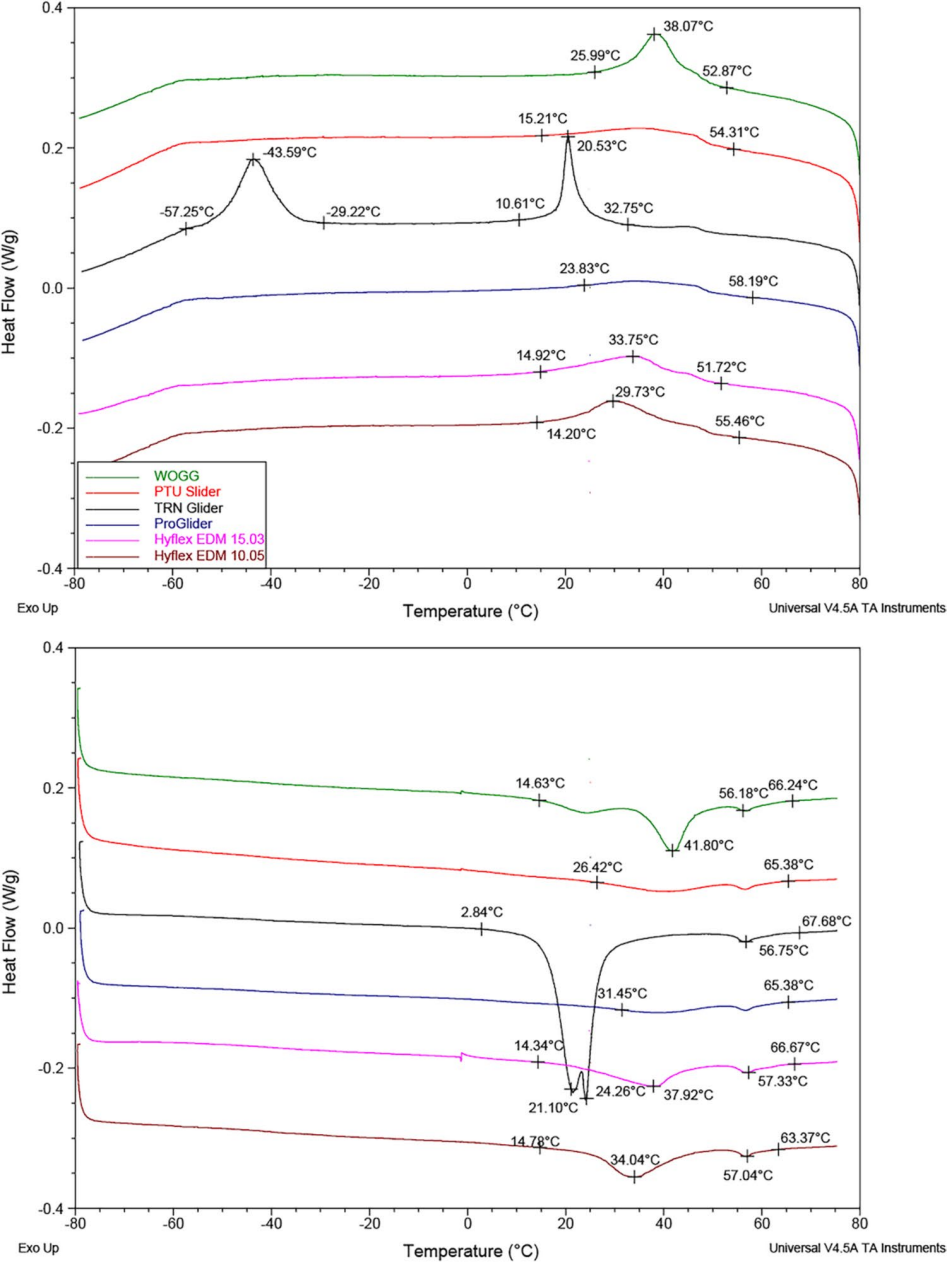
Scanning electron microscopic images of the fractured surfaces (first column) revealed varying degrees of roughness and patterns typical of torsional overload. The cyclic fatigue test images display rough surfaces with striations and multiple initiation points, indicative of cyclic loading failure (× 50 to × 350 magnification)

Scanning electron microscopic images were obtained on fractured surfaces after cyclic and torsional strength tests to show cross-sectional shape and confirm fracture types on 2 randomly selected instruments from each group at 50×–350× magnifications (JEOL, JSM-5200, Tokyo, Japan) (Fig. 3).

#### Differential scanning calorimetry

Differential scanning calorimetry (DSC) analyses were performed on 5 cut instruments from each group into 2 segments with 3–4 mm in length, which were further weighted (6 ± 1 mg) using a slow speed water-cooled diamond saw. The differential scanning calorimeter (DSC Q2000, TA Instruments LLC, New Castle, USA) with argon gas and liquid nitrogen used for the cooling process was conducted with a temperature range from −80 °C to 80 °C with 10 °C/min heating rate triplicate for each specimen.

#### Microhardness test

For microhardness measurement, sample size determination indicated 3 specimens per group was adequate based on the effect size of a previous study (1.83) [21]. Surface microhardness values were tested on 4 instruments, which were longitudinally embedded in resin, and surfaces were polished and ultrasonically cleaned. Vickers hardness was measured using a microhardness tester (HMV-G Shimadzu, Tokyo, Japan) equipped with a Vickers indenter under 100 g load and 15 s contact time on D2, D4, D6, D10, and D14 levels.

#### SEM energy dispersive spectrometry

Using SEM energy dispersive spectrometry, Ni and Ti content (%wt) was determined from the center of specimens’ polished surfaces after the instrument was longitudinally embedded to resin (*n* = 5), with 15 kV accelerated voltage, vacuum pressure 9.6 × 10^–5^ Pa, and X300 magnification using X-Max 80 detector (Oxford Instruments Comp., Buckinghamshire, UK).

### Statistical analysis

The conformity normal distribution of the data was assessed using the Shapiro–Wilk test. The data that did not fit the normal distribution (*p* < 0.05) were analyzed using the Kruskal–Wallis *H* and Mann–Whitney *U* tests with Bonferroni correction. The data following the normal distribution (*p* > 0.05) was analyzed with one-way analysis of variance and post-hoc Tukey tests. For cyclic fatigue tests, Weibull analyses were conducted to estimate **a** 99% survival rate. All statistical analyses were conducted with a significance level of 5% using the SPSS (V.21, Chicago, IL, USA).

## Results

### Design

The helix angles ranged from 17.12° to 24.14°, with TN Glider showing the greatest angle and HF EDM 15.03 the lowest (Table 1). The blade numbers were similar between 19 and 21, except with both HF instruments having 16 blades each. All instruments had non-active tips with different designs. No major defects or deformations were observed in any of the instruments. SEM examination revealed minor irregularities, such as metal rollover on cutting edges in all instruments. Surface finishing differed in HF files, while all of the remaining files had milling grooves perpendicular to their long axis (Fig. 2). HF EDMs showed crater-like surfaces along the length of the file due to ED machining. At × 300 magnification, these crater-like structures seem less common in HF 15.03 than in HF 10.05; however, at × 500 magnification, both instrument surfaces were similar. The deviations of the measuring lines were within the limits in PTU Slider, PG and TN Glider, while deviations greater than 0.1 mm were detected in WOGG and HF EDM files.

**Table 1:** Helix angle, blade number, number of defects, mean and standard deviation values for distance of measuring lines from instrument tip, surface Vickers Microhardness, and Ni/Ti ratio values of the tested glide path instruments

	Position of measuring lines (mm)				Helix angle (°)	Blade number	Defects/deformations	Vickers microhardness (HVN)	Ni/Ti ratio (wt%)
	18 mm	19 mm	20 mm	22 mm					
ProTaper Ultimate Slider	17.93 ± 0.08	18.92 ± 0.09	19.98 ± 0.04	22.00 ± 0.00	23.13	19	0	498.40 ± 48.71^a^	1.30
ProGlider	-	-	19.93 ± 0.11	21.95 ± 0.07	20.92	21	0	524.60 ± 94.13^a^	1.28
TruNatomy Glider	17.97 ± 0.06	18.99 ± 0.03	19.98 ± 0.04	21.98 ± 0.04	24.14	22	0	478.73 ± 74.17^a^	1.20
WaveOne Gold Glider	17.91 ± 0.09	**18.89 ± 0.07**	19.90 ± 0.11	21.91 ± 0.07	21.51	21	0	524.60 ± 94.13^a^	1.28
Hyflex EDM 10.05	**17.79 ± 0.07**	**18.84 ± 0.08**	**19.81 ± 0.04**	21.97 ± 0.00	20.39	16	0	541.00 ± 76.63^a^	1.22
Hyflex EDM 15.03	**17.84 ± 0.08**	18.94 ± 0.08	19.94 ± 0.05	**21.88 ± 0.07**	17.12	16	0	526.86 ± 73.52^a^	0.91

In the measuring lines, deviations greater than 0.1 mm from reference points were identified with bold letters

### Mechanical analysis

In both static and dynamic models**,** WOGG and PG had the greatest time to fracture values with no significant difference between them (*p* > 0.05) (Table 2). TN Glider showed significantly lower TF values compared to WOGG, PG, PTU S (*p* < 0.05). PTU Slider and HF EDM 15.03 instruments showed similar cyclic fatigue strength (*p* > 0.05). In the dynamic cyclic fatigue tests, the Weibull modulus ranged from 2.98 to 6.58, with HF EDM 10.05 exhibiting the highest value, while in static tests, it ranged from 2.68 to 10.80, with PTU Slider having the highest value. Fragment lengths did not differ in both cyclic fatigue models (*p* > 0.05).

**Table 2:** Mechanical test results regarding cyclic fatigue, torsional, bending and buckling resistance of tested instruments

	Dynamic cyclic fatigue resistance				Static cyclic fatigue resistance				Torsional resistance		Bending resistance (gf)	Buckling resistance (gf)
	Time to fracture (s)	Weibull modulus	*R*^2^	Time required for 99% survival	Time to fracture (s)	Weibull modulus	*R*^2^	Time required for 99% survival	Ultimate torsional resistance	Angular deflection		
ProTaper Ultimate Slider	385.91 ± 87.8^a^	4.03	0.96	614	579.17 ± 56.8^a^	10.80	0.94	696.49	0.56 ± 0.06^a^	283.0 ± 23.70^a^	**0.20 ± 0.02**^**a**^	**0.74 ± 0.23**^**a**^
ProGlider	**1090.25 ± 260.6**^**b**^	4.13	0.93	1737	**1114.92 ± 223.3**^**b**^	5.20	0.95	1616.56	0.49 ± 0.02^a^	268.0 ± 32.49^a^	0.15 ± 0.02^b^	0.64 ± 0.14^ab^
TruNatomy Glider	66.08 ± 15.2^c^	4.76	0.95	100	184.58 ± 33.2^c^	5.62	0.94	260.29	0.60 ± 0.11^ab^	412.0 ± 82.08^b^	0.07 ± 0.02^c^	0.38 ± 0.17^bc^
WaveOne Gold Glider	**988.91 ± 182.1**^**b**^	5.48	0.97	1406	**1285.08 ± 185.0**^**b**^	7.39	0.93	1678.44	0.52 ± 0.09^a^	487.0 ± 70.80^c^	0.08 ± 0.01^c^	0.31 ± 0.18^c^
Hyflex EDM 10.05	141.91 ± 50.0^c^	6.58	0.92	266	441.92 ± 173.1^c^	2.68	0.89	876.89	**0.68 ± 0.08**^**bc**^	370.0 ± 54.35^b^	0.07 ± 0.02^c^	0.45 ± 0.14^bc^
Hyflex EDM 15.03	545.16 ± 98.9^a^	2.98	0.90	741	666.08 ± 164.9^a^	4.47	0.74	1027.03	**0.79 ± 0.03**^**c**^	363.0 ± 54.90^b^	0.09 ± 0.02^c^	0.64 ± 0.28^ab^

Different superscript letters in the same column indicate statistically significant difference among different glide path instruments (*p* < .05)

Bolded values represent the best-performing instrument(s) for each specific test

Ultimate torsional resistance of HF EDM 15.03 was significantly greater than PTU Slider, PG, WOGG and TN Glider (*p* < 0.05) and similar to HF EDM 10.05 (*p* > 0.05). No significant difference was found between HF EDM 10.05 and TN Glider (*p* > 0.05). WOGG showed significantly greater angular deflection values among all groups (*p* < 0.05). Angular deflection values of TN Glider and HF EDM instruments were similar (*p* > 0.05). The lowest angular deflection values were detected in PTU Slider and PG groups, with no significant difference between them (*p* > 0.05).

The highest bending resistance was detected in PTU Slider among the tested instruments (*p* < 0.05). The bending resistance of TN Glider, WOGG, and HF EDM instruments were significantly lower than that of PG instruments (*p* < 0.05). Buckling resistance of PTU Slider, PG and HF EDM 15.03 was significantly higher than those of TN Glider, WOGG and HF EDM 10.05 (*p* < 0.05).

### Metallurgical analysis

**Fig. 4:**
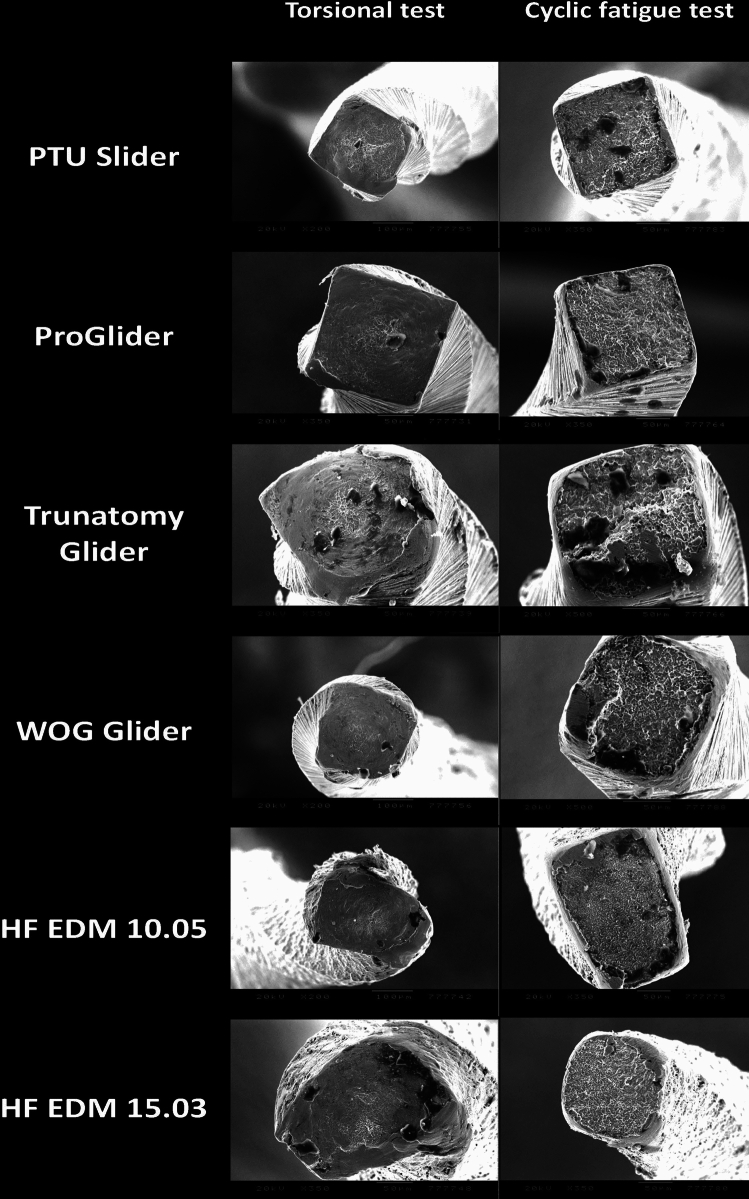
DSC plots showing the phase transformation temperatures during cooling (upper chart) and heating process (lower chart). All instruments had a mixture of R-phase and martensitic crystallographic arrangement at intracanal temperature except TRN Glider showed completed austenitic transformation

The DSC curves showing phase transformation behavior during cooling and heating processes are provided in Fig. 4. Upper peak indicates the exothermic reaction of martensitic transformation from austenite phase. During cooling, WOGG showed a single prominent peak, while TN Glider exhibited 2 prominent peaks, which suggests a two-stage phase transformation from austenite to martensite, including R-phase. Martensitic transformation start (*M*_s_) temperatures were similar among instruments apart from the TN Glider, which had *R*_s_ temperature at 32 °C. During the heating cycle, the *A*_f_ temperature of the TN Glider was lower than those of the remaining instruments and body temperature. Elemental composition regarding the Ni/Ti ratio of the instruments is presented in Table 1. PTU Slider and PG consisted of Ni and Ti, whereas the remaining instruments had O, Ni and Ti. While all instruments showed a peak for the C, albeit small, all instruments except the PTU Slider also showed low peaks for the Si. Vickers Microhardness values of instrument surfaces were similar among the instruments (*p* > 0.05).

## Discussion

Glide path instruments serve as the first NiTi files used in root canal treatments, primarily designed to navigate and create a smooth path through curved and narrow canals. Due to their role in negotiating complex anatomy, they endure significant flexural and torsional stresses. The glide path instruments commonly encounter obliterated canals in the middle third, while in the apical third, there are acute apical curvatures, which may not be detected with preoperative periapical radiographs. The anatomical difficulty of unnegotiated canals requires glide path instruments to have significant flexibility and the ability to withstand repeated bending and torsional stresses. This study employed a multimethod approach to assess the design, mechanical properties, and metallurgical characteristics of novel and commonly used glide path instruments. Since significant differences were observed in terms of these parameters, the null hypothesis was rejected.

The multimethod analysis of glide path instruments is clinically important as it provides a holistic understanding of their mechanical performance by integrating design and metallurgical insights. By evaluating static and dynamic cyclic fatigue, torsional resistance, bending resistance, and buckling resistance following strict international guidelines, clinicians can make informed choices regarding instrument selection for specific clinical scenarios. This comprehensive evaluation helps identify instruments with optimal flexibility, strength, and durability, ensuring safer and more efficient root canal procedures. Moreover, understanding the impact of design and metallurgy on mechanical characteristics allows for the selection of instruments that can effectively navigate complex root canal anatomies while minimizing the risk of iatrogenic errors. However, it is important to interpret laboratory test results with caution when applying them to clinical settings.

None of the tested instruments exhibited major defects or deformations, though all displayed distinct design features along with minor imperfections, such as metal rollovers on the blades. The measuring line positions were accurate in PTU Slider, PG and TN Glider. HF EDM instruments differed from the others in surface finishing due to EDM manufacturing, which results in a characteristic crater-like surface caused by localized melting and evaporation [22]. EDS analysis detected traces of C and Si, likely due to environmental contamination [23]. Instruments that underwent EDM or post-machining heat treatment contained an oxide layer (O element), except for PG, which lacked this layer. Ni/Ti ratio ranged between 0.91 and 1.30 wt% among instruments; the highest was detected in the PTU Slider. The surface microhardness values were similar among instruments and greater than dentine microhardness [24]. Microhardness can be measured from the surface of the instrument or more easily by exposing a flat core area by cutting and grinding. Surface microhardness values were obtained in the present study, because rather than eliminating the oxide layer, surface indentation has been suggested to be evaluated as an indicative of cutting ability [25]. Although a linear relationship between Ni content and surface hardness has been demonstrated in the past [26], the surface hardness values in this study were inconsistent with the Ni content. These discrepancies may be attributed to variations in the manufacturing processes, as reported by Zinelis et al. [21]. Furthermore, it has been shown that austenite finish temperatures influence the mechanical properties of NiTi files more than their atomic composition [21]. The Ni/Ti ratio ranged between 0.91 and 1.30 wt%, with PTU Slider exhibiting the highest Ni content. Since higher Ni content is associated with increased flexibility, this could explain PTU Slider’s superior bending and buckling resistance, making it a potentially safer choice for navigating severely curved or constricted canals [21, 27]. However, instruments with increased Ni content may also show reduced cutting efficiency, which should be considered when selecting files for initial glide path creation.

In the present study, the cyclic fatigue tests were conducted using both static and dynamic test models using the same block with an acute apical curvature. Dynamic test models are known to extend instrument lifespan by preventing microcracks from propagating into fractures due to the continuous change in compression–tension areas. In this study, intracanal temperature was simulated in both models, but the static test block was embedded in a heated water bath, while the dynamic test model tested instruments in air. Regardless of the instrument type, instruments in the static model demonstrated longer lifespans than those in the dynamic model. This finding is unusual and not typically reported in previous studies conducted at room temperature and in air [28]. The difference might be attributed to the use of a simulated canal with acute apical curvature, different test environments (air vs. liquid) and simulation of intracanal temperature instead of room temperature [29, 30]. Intergroup comparison among different glide path files yielded similar results in both models. PG and WOGG showed the highest cyclic fatigue resistance, followed by HF EDM 15.03, PTU Slider. TN Glider and HF EDM 10.05 showed significantly lower cyclic fatigue resistance values. Since HF EDM instruments share the same metallurgy as seen in DSC curves, the difference between their cyclic fatigue resistance could be caused by the greater taper of 10.05 file.

The phase transformation behavior of TN Glider differed from the other instruments, displaying a two-step austenite-to-R-phase transformation at body temperature and room temperature, followed by an R-phase-to-martensite transformation below 0 °C. Therefore, ambient temperature during the cyclic fatigue tests might reveal lower cyclic fatigue resistance, since TN Glider had an austenitic state in body temperature, while the remaining instruments had crystallographic arrangements with more martensitic and/or R-phases. This finding suggests that TN Glider may be more prone to fracture during prolonged use in curved canals, particularly in cases requiring extended negotiation or multiple instrument insertions. Clinicians should be aware of this when selecting instruments for challenging anatomy and may need to consider alternative glide path files with superior fatigue resistance for such cases. Similar findings were reported in a recent study that the TN instrument showed lower cyclic fatigue resistance in accordance with lower austenite to R-phase transformation temperature [31]. These findings emphasize the clinical importance of a multimethod approach in evaluating glide path instruments to ensure their optimal selection and use in endodontic procedures.

The necessity of simulation of the ambient temperature as body or intracanal temperature during mechanical tests has also been discussed for being not precise and depending on many variables [25]. These variables are the instrument’s exposed surface area, exposure time and change of intracanal temperatures during different phases of root canal preparation [25]. In the present study, a thermocouple was inserted into a hole on the artificial block reaching the same region as the instruments were placed and confirmed the simulation of body temperature in the center of the block itself. A previous study confirmed this temperature range by measuring file temperatures after removal from root canals, reporting a range between 30.8 °C and 32.5 °C prior to cyclic fatigue testing [32].

NiTi instruments undergo heat treatment during manufacturing, which alters their phase transformation temperatures to counteract work hardening effects. Post-manufacturing heat treatments further modify phase transformation temperatures to achieve specific mechanical properties. Miyara et al. reported that heat treatments at 300 °C and 600 °C keep NiTi alloys primarily in the austenitic phase at body temperature, whereas treatments at 400–450 °C result in a more flexible mixed-phase material [33]. Because proprietary details of these heat treatments remain undisclosed by manufacturers, DSC plots serve as an essential tool for correlating phase transformation behavior with mechanical properties.

Recently, the concept of a “service temperature range” has been introduced to describe mechanical properties of NiTi alloys in the light of DSC plots ranging between the room and body temperature [25]. Examination of the heating curves from the DSC plots between 20 °C and 37 °C revealed distinct transformation behaviors among the tested instruments. WOGG and PTU Slider remained predominantly in the martensitic phase throughout this range, which correlates with their high cyclic fatigue resistance.

ProGlider (PG) and HyFlex EDM instruments also began in the martensitic phase at room temperature but gradually transitioned to a mixed-phase state as the temperature increased. HF EDM instruments exhibited this transformation earlier, between 25 °C and 34 °C, becoming more austenitic above 34 °C.

In contrast, TN Glider demonstrated a different transformation profile. Between 20 °C and 33 °C, it was predominantly in a mixed R-phase/austenite state, completing the transition to a fully austenitic phase beyond 33 °C. This crystallographic constitution at intracanal temperature may explain its significantly lower cyclic fatigue resistance compared to the other instruments, which retained martensitic or mixed-phase structures during testing. In the present study, the torsional resistance of glide path instruments was tested according to international standards [34] at room temperature, where all instruments were in a martensitic state. The findings of this study are in accordance with a recent multimethod study that compared ProGlider and PTU Slider instruments and reported similarities in terms of torsional resistance [35]. HF EDM instruments showed superior torsional resistance, while TN Glider and WOGG displayed higher angular deflection values, which is regarded as a clinically detectable warning that the file reaches the plastic limits [36]. PTU Slider showed good torsional and flexural resistance accompanied by the highest bending and buckling resistance among the tested instruments. Buckling resistance is specifically important for glide path instruments that might encounter a light pressure during canal negotiation. In the bending test, the TN Glider, WOGG, and HF EDM instruments were the most flexible ones. The increase in flexibility is associated with high cyclic fatigue resistance in instruments, such as WOGG and HF, while for the TN Glider, it appears to be related to a thinner maximum flute diameter.

By assessing the design, mechanical properties, and metallurgical characteristics of glide path instruments, this study provides clinically relevant insights for selecting the most suitable instrument in various endodontic scenarios, where a glide path preparation is required. The multimethod approach conducted in this study enabled a more comprehensive assessment of glide path instruments’ mechanical strengths and behaviors. The lack of tests evaluating the cutting efficiency, time required for reaching length and negotiation and shaping ability are the limitations of this study, which should be evaluated in future studies.

## Conclusion

Mechanical tests indicate that instruments such as the WOGG and PG offer superior cyclic fatigue resistance, while the HF EDM and TN Glider exhibit the highest torsional resistance. The PTU Slider stands out for its high buckling resistance combined with relatively higher torsional and cyclic fatigue resistance values. These mechanical properties are closely related to phase transformation behavior. In addition, the WOGG and TN Glider demonstrated higher angular deflection angles.

## Data Availability

Data will be shared upon reasonable request from the corresponding author.
